# A triclinic polymorph of (−)-(*S*)-*N*-benzyl-2-[(*R*)-6-fluoro­chroman-2-yl]-2-hy­droxy­ethanaminium bromide

**DOI:** 10.1107/S1600536813030377

**Published:** 2013-11-20

**Authors:** Yoann Rousselin, Hugo Laureano, Alexandre Clavel

**Affiliations:** a Institut de Chimie Moleculaire de l’Universite de Bourgogne - ICMUB, UMR CNRS 6302, Universite de Bourgogne, 9, Av. Alain Savary, 21078 Dijon CEDEX, France; b CordenPharma-Synkem, 47 rue de Longvic, 21301 Chenove, France

## Abstract

The title salt, C_18_H_21_FNO_2_^+^·Br^−^, determined at 115 K, crystallizes in the triclinic space group *P*1. The previously reported polymorph occurs in the monoclinic space group *P*2_1_ and has two independent mol­ecules in the asymmetric unit [Peeters *et al.* (1993 ▶). *Acta Cryst.* C**49**, 2157–2160]. In the title molecule, the pyran rings adopt half-chair conformations. The absolute configuration is *S* for the hy­droxy-bearing C atom and *R* for the asymmetric C atom in the di­hydro­pyran unit. In the crystal, the components are linked by N—H⋯Br and O—H⋯Br hydrogen bonds, forming chains along the *c*-axis direction. The crystal studied was refined as an inversion twin.

## Related literature

For the synthesis of the enanti­opure title product, see: Jas *et al.* (2011 ▶). For studies of related isomers, see: Cini *et al.* (1990 ▶); Tuchalski *et al.* (2006 ▶, 2008 ▶); Rousselin *et al.* (2012 ▶). for the monoclinic polymorph, see: Peeters *et al.* (1993 ▶). The title compound is a key inter­mediate in the synthesis of the beta blocker dl-nebivolol [systematic name: 1-(6-fluoro­chroman-2-yl)-{[2-(6-fluoro­chroman-2-yl)-2-hy­droxy-eth­yl]amino}­ethanol. For the pharmacological properties of nebivolol, see: Van Lommen *et al.* (1990 ▶). For puckering parameters, see: Cremer & Pople (1975 ▶). For background to polymorphism, see: Bernstein (2002 ▶).
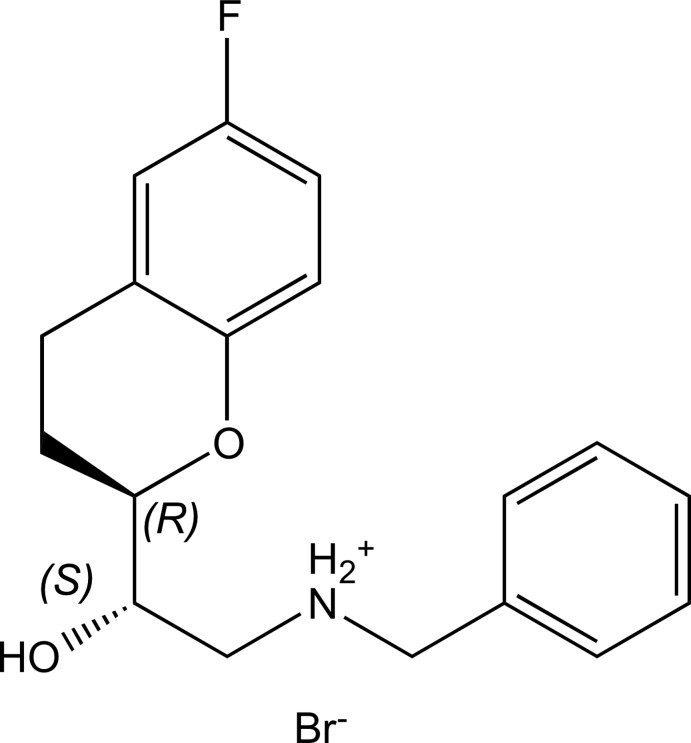


## Experimental

### 

#### Crystal data

C_18_H_21_FNO_2_^+^·Br^−^
*M* *_r_* = 382.27
Triclinic, 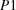

*a* = 4.9248 (2) Å
*b* = 5.5117 (2) Å
*c* = 16.3894 (7) Å
α = 83.721 (2)°
β = 89.038 (2)°
γ = 86.765 (2)°
*V* = 441.48 (3) Å^3^
*Z* = 1
Mo *K*α_1_ radiation
μ = 2.35 mm^−1^
*T* = 115 K
0.25 × 0.2 × 0.2 mm

#### Data collection

Nonius KappaCCD diffractometer with APEXII detector
Absorption correction: multi-scan (*SADABS*; Bruker, 2012 ▶) *T*_min_ = 0.61, *T*_max_ = 0.74
10328 measured reflections
3903 independent reflections
3886 reflections with *I* > 2σ(*I*)
*R*_int_ = 0.020

#### Refinement

*R*[*F* ^2^ > 2σ(*F* ^2^)] = 0.021
*wR*(*F* ^2^) = 0.052
*S* = 1.11
3903 reflections
210 parameters
3 restraints
H-atom parameters constrained
Δρ_max_ = 0.37 e Å^−3^
Δρ_min_ = −0.18 e Å^−3^
Absolute structure: Flack (1983 ▶); refined as an inversion twin
Absolute structure parameter: 0.013 (7)

### 

Data collection: *APEX2* (Bruker, 2012 ▶); cell refinement: *SAINT* (Bruker, 2012 ▶); data reduction: *SAINT*; program(s) used to solve structure: *SHELXS97* (Sheldrick, 2008 ▶); program(s) used to refine structure: *SHELXL97* (Sheldrick, 2008 ▶); molecular graphics: *OLEX2* (Dolomanov *et al.*, 2009 ▶); software used to prepare material for publication: *OLEX2*.

## Supplementary Material

Crystal structure: contains datablock(s) I. DOI: 10.1107/S1600536813030377/gw2140sup1.cif

Structure factors: contains datablock(s) I. DOI: 10.1107/S1600536813030377/gw2140Isup2.hkl

Supplementary material file. DOI: 10.1107/S1600536813030377/gw2140Isup3.cdx

Supplementary material file. DOI: 10.1107/S1600536813030377/gw2140Isup4.cml

Additional supplementary materials:  crystallographic information; 3D view; checkCIF report

## Figures and Tables

**Table 1:** Hydrogen-bond geometry (Å, °)

*D*—H⋯*A*	*D*—H	H⋯*A*	*D*⋯*A*	*D*—H⋯*A*
N1—H1*A*⋯Br1^i^	0.99	2.40	3.306 (2)	152
N1—H1*B*⋯Br1^ii^	0.99	2.30	3.258 (2)	162
O2—H2*A*⋯Br1^i^	0.84	2.47	3.2198 (19)	149

Symmetry codes: (i) 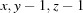; (ii) 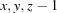.

**Fig. 1.**
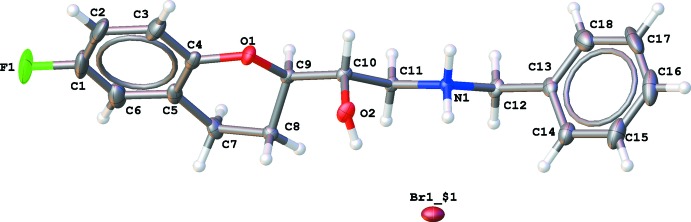
View of the molecular structure of (I) with 50% probability displacement ellipsoids for the non-hydrogen atoms.

**Fig. 2.**
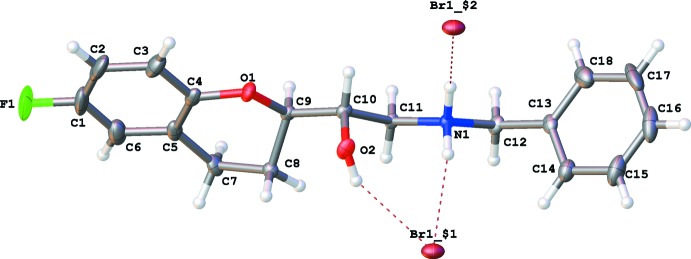
View of the hydrogen-bonding in (I). Dashed lines indicate O—H···Br and N—H···Br hydrogen bonds.

**Fig. 3.**
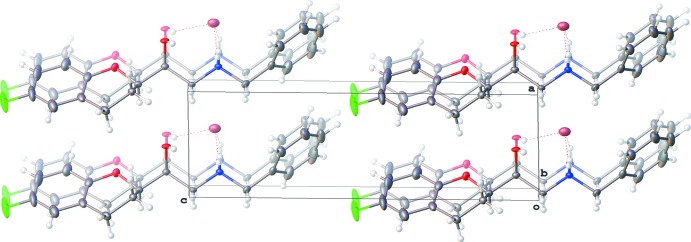
Packing features in (I).

## supplementary crystallographic information

### 1. Introduction

The asymmetric unit of title compound is a key building block for the synthesis of *dl*-nebivolol. This active pharmaceutical ingredient is a highly cardioselective vasodilatory *β*-receptor blocker used in treatment of hypertension. The chemical structure of nebivolol contains four asymmetric carbon atoms (chiral centers), the combination of all the centers results in 16 theoretical isomers and the total number of isomeric structures is reduced to 10 due to the symmetry plane through the N atom of the molecule. All isomeric structures are currently known (Tuchalski *et al.* (2006), Rousselin *et al.* (2012)). This paper confirms absolute configuration of one possible amino inter­mediate formed during the synthesis of *dl*-nebivolol. This structure is a polymorphic form (Bernstein, 2002) of a previous structure determined by Peeters *et al.* (1993).

### 3. Synthesis and crystallization

2-chloro-1-(6-fluoro-chroman-2-yl)-1-ethanol were prepared as enanti­opure products in order to obtain the 2-benzyl­amino-1-(6-fluoro-chroman-2-yl)-1-ethanol by addition of benzyl­amine (Jas *et al.* (2011)). A subsequent addition of (*R*)-2-chloro-1-((*S*)-6-fluoro-chroman-2-yl)-1-ethanol would then be used to yield the corresponding protected nebivolol. The crude 2-benzyl­amino-1-(6-fluoro-chroman-2-yl)-1-ethanol was then recrystallized at 60°C in a mixture of ethanol and aqueous hydro­bromic acid.

The X-ray, mass spectrometry and NMR analyzes was recorded in the "Pôle Chimie Moléculaire", the technological platform for chemical analysis and molecular synthesis (http://www.wpcm.fr) which relies on the Institute of the Molecular Chemistry of University of Burgundy and Welience"TM", a Burgundy University private subsidiary. The analytical results concerning identity (NMR and optical rotation) and purity (HPLC and chiral HPLC) are listed below. 1H and 13C NMR measurements were performed in deuterated DMSO on Bruker Avance III, recorded at 300 MHz and 75.5 MHz, respectively. DMSO-d6 has been used as inter­nal reference. Chemical shifts (δ) and coupling constants are reported respectively in p.p.m. and hertz (Hz). The optical rotation was measured using a UV Visible Perkin Elmer Lambda 12, polarimeter at 589 nm. High-resolution mass spectrometry (HRMS) was performed in ESI a positive mode. The infrared spectrum (IR) was generated by ATR using a Spectrometer Infrared Avatar 370. A scan range of 4000 - 400 cm^-1^ was used.

(*S*)-2-benzyl­amino-1-((*R*)-6-fluoro-chroman-2-yl)-1-ethanol characterization:

δ(^1^H, DMSO-d6, 300 MHz, ppm): 1.69 (1H, m); 2.04 (2H, m); 2.58 (1H, m); 2.74 (3H, m); 3.65 – 3.78 (1H, m); 3.73 (2H, s); 3.88 (1H, m); 5.00 (1H, bs); 6.68 (1H, m); 6.88 (2H, m); 7.17 – 7.37 (5H, m).

δ(^13^C DMSO-d6, 75.47 MHz, ppm): 22.1; 23.8; 51.3; 53.0; 70.7; 77.3; 113.5 (d, 22.5 Hz); 115.2 (d, 22.5 Hz); 117.2 (d, 8.3 Hz); 123.8 (d, 7.5 Hz); 126.4; 127.8; 128.0; 140.9; 150.6 (d, 2.3 Hz); 155.7 (d, 234 Hz).

HRMS (ESI) for C_18_H_21_FNO_2_[M+H]^+^ m/z =302.15508, found m/z = 302.1537.

IR (cm^-1^) 3137, 2821, 1489, 1215, 810.

### 4. Refinement

Crystal data, data collection and structure refinement details are summarized in Table 1. Anisotropic thermal parameters were used for non-hydrogen atoms. All H atoms, on carbon atom or oxygen atom, were placed at calculated positions using a riding model with C—H = 1 Å (methine), 0.99 Å (methyl­ene), 0.95 Å (aromatic), N—H = 0.99 Å or O—H = 0.84 Å with Uiso(H) = 1.2Ueq(CH), Uiso(H) = 1.2Ueq(CH_2_), Uiso(H) = 1.2Ueq(NH) or Uiso(H) = 1.5Ueq(OH). TWIN/BASF refinement type was used to determine absolute configuration from anomalous scattering using the Flack method.

### 5. Results and discussion

The asymmetric unit of the title compound, C_18_H_21_FNO_2_^+^.Br^-^, contains one molecule. Contrary to the previous structure (Peeters *et al.*, 1993) which crystallized in monoclinic with *P*2_1_ symmetry, we found a polymophic form which crystallize in triclinic with *P*1 symmetry. The overlay of the molecules obtained in two different crystal systems clearly shows that they possess the same conformation with RMSD of 0.1329 Å, 0.1088 Å and a maximum deviation of 0.2823 Å, 0.1789 Å. The pyran rings adopt half-chair conformations with total puckering amplitutdes QT of 0.5041 (28) (with Θ = 129.10 (33)° and φ = 84.59 (39)°) (Cremer & Pople, (1975)). The protonation of the amine is confimed by the distance C11—N1 and C12—N1 of 1.502 (3) and 1.513 (4) respectively. The structure is stabilized by a network of hydrogen bonds between N, O and Br atoms. The absolute configuration is R for the asymmetric C atom in the di­hydro­pyran ring and S for the hydroxyl-bearing C atom. Chains are formed in the c axis.

Concerning the crystal packing features, each aromatic group are parallel unlike the previously determined structure (Peeters *et al.*, 1993) wherein aromatic rings between two adjacent molecules possess dihedral angle close to 35° and 55° respectively.

### Crystal data

**Table:**

C_18_H_21_FNO_2_^+^·Br^−^	*V* = 441.48 (3) Å^3^
*M**_r_* = 382.27	*Z* = 1
Triclinic, *P*1	*F*(000) = 196
*a* = 4.9248 (2) Å	*D*_x_ = 1.438 Mg m^−^^3^
*b* = 5.5117 (2) Å	Mo *K*α_1_ radiation, λ = 0.71073 Å
*c* = 16.3894 (7) Å	µ = 2.35 mm^−^^1^
α = 83.721 (2)°	*T* = 115 K
β = 89.038 (2)°	Prism, clear light colourless
γ = 86.765 (2)°	0.25 × 0.2 × 0.2 mm

### Data collection

**Table:**

Nonius KappaCCD diffractometer with APEXII detector	3903 independent reflections
Radiation source: X-ray tube, Siemens KFF Mo 2K-180	3886 reflections with *I* > 2σ(*I*)
Graphite monochromator	*R*_int_ = 0.020
Detector resolution: 9 pixels mm^-1^	θ_max_ = 27.6°, θ_min_ = 3.7°
CCD rotation images, thick slices scans	*h* = −6→6
Absorption correction: multi-scan (*SADABS*; Bruker, 2012)	*k* = −7→6
*T*_min_ = 0.61, *T*_max_ = 0.74	*l* = −21→21
10328 measured reflections	

### Refinement

**Table:**

Refinement on *F*^2^	Hydrogen site location: inferred from neighbouring sites
Least-squares matrix: full	H-atom parameters constrained
*R*[*F*^2^ > 2σ(*F*^2^)] = 0.021	*w* = 1/[σ^2^(*F*_o_^2^) + (0.0317*P*)^2^ + 0.0488*P*] where *P* = (*F*_o_^2^ + 2*F*_c_^2^)/3
*wR*(*F*^2^) = 0.052	(Δ/σ)_max_ < 0.001
*S* = 1.11	Δρ_max_ = 0.37 e Å^−^^3^
3903 reflections	Δρ_min_ = −0.18 e Å^−^^3^
210 parameters	Absolute structure: Flack (1983); refined as an inversion twin
3 restraints	Absolute structure parameter: 0.013 (7)
0 constraints	

### Special details

**Table:**

Geometry. All e.s.d.'s (except the e.s.d. in the dihedral angle between two l.s. planes) are estimated using the full covariance matrix. The cell e.s.d.'s are taken into account individually in the estimation of e.s.d.'s in distances, angles and torsion angles; correlations between e.s.d.'s in cell parameters are only used when they are defined by crystal symmetry. An approximate (isotropic) treatment of cell e.s.d.'s is used for estimating e.s.d.'s involving l.s. planes.
Refinement. Refined as a 2-component inversion twin.

### Fractional atomic coordinates and isotropic or equivalent isotropic displacement parameters (Å^2^)

**Table:**

	*x*	*y*	*z*	*U*_iso_*/*U*_eq_	
C1	−0.0450 (9)	0.1145 (8)	0.4454 (2)	0.0432 (10)	
C2	0.1300 (8)	0.3000 (9)	0.4294 (2)	0.0438 (11)	
H2	0.1974	0.3810	0.4725	0.053*	
C3	0.2056 (7)	0.3656 (7)	0.3481 (2)	0.0336 (7)	
H3	0.3260	0.4932	0.3348	0.040*	
C4	0.1030 (6)	0.2425 (5)	0.28630 (16)	0.0208 (5)	
C5	−0.0712 (6)	0.0542 (6)	0.30396 (17)	0.0229 (6)	
C6	−0.1462 (7)	−0.0085 (7)	0.3862 (2)	0.0350 (8)	
H6	−0.2666	−0.1356	0.4003	0.042*	
C7	−0.1764 (6)	−0.0806 (7)	0.2368 (2)	0.0220 (6)	
H7A	−0.3673	−0.0241	0.2246	0.026*	
H7B	−0.1721	−0.2580	0.2551	0.026*	
C8	−0.0012 (6)	−0.0345 (6)	0.15966 (19)	0.0155 (6)	
H8A	0.1775	−0.1248	0.1676	0.019*	
H8B	−0.0911	−0.0932	0.1125	0.019*	
C9	0.0379 (5)	0.2363 (5)	0.14248 (15)	0.0135 (5)	
H9	−0.1448	0.3254	0.1390	0.016*	
C10	0.2004 (5)	0.3138 (5)	0.06472 (15)	0.0142 (5)	
H10	0.2312	0.4917	0.0642	0.017*	
C11	0.0363 (5)	0.2836 (6)	−0.01119 (18)	0.0122 (6)	
H11A	−0.0166	0.1125	−0.0092	0.015*	
H11B	−0.1318	0.3911	−0.0120	0.015*	
C12	0.0371 (6)	0.3492 (6)	−0.1649 (2)	0.0174 (7)	
H12A	−0.0300	0.1845	−0.1681	0.021*	
H12B	−0.1225	0.4662	−0.1633	0.021*	
C13	0.2086 (6)	0.4206 (5)	−0.23948 (16)	0.0186 (5)	
C14	0.4109 (6)	0.2578 (6)	−0.26486 (18)	0.0263 (6)	
H14	0.4397	0.1012	−0.2349	0.032*	
C15	0.5715 (8)	0.3220 (8)	−0.3336 (2)	0.0394 (8)	
H15	0.7117	0.2107	−0.3494	0.047*	
C16	0.5283 (9)	0.5446 (9)	−0.3784 (2)	0.0438 (10)	
H16	0.6358	0.5868	−0.4260	0.053*	
C17	0.3274 (12)	0.7082 (9)	−0.3541 (2)	0.0481 (13)	
H17	0.2989	0.8634	−0.3851	0.058*	
C18	0.1655 (10)	0.6482 (8)	−0.2847 (2)	0.0332 (10)	
H18	0.0277	0.7615	−0.2685	0.040*	
N1	0.2011 (4)	0.3481 (4)	−0.08768 (13)	0.0128 (4)	
H1A	0.3584	0.2287	−0.0893	0.015*	
H1B	0.2725	0.5118	−0.0859	0.015*	
O1	0.1885 (4)	0.3208 (4)	0.20789 (11)	0.0191 (4)	
O2	0.4597 (3)	0.1871 (4)	0.06423 (12)	0.0191 (4)	
H2A	0.4477	0.0574	0.0423	0.029*	
F1	−0.1223 (7)	0.0501 (6)	0.52531 (13)	0.0673 (9)	
Br1	0.56053 (2)	0.82061 (2)	0.92529 (2)	0.02213 (8)	

### Atomic displacement parameters (Å^2^)

**Table:**

	*U*^11^	*U*^22^	*U*^33^	*U*^12^	*U*^13^	*U*^23^
C1	0.055 (2)	0.058 (3)	0.0135 (14)	0.0171 (19)	0.0071 (14)	−0.0006 (14)
C2	0.058 (3)	0.055 (3)	0.0186 (15)	0.016 (2)	−0.0064 (19)	−0.0170 (16)
C3	0.0379 (18)	0.042 (2)	0.0223 (15)	0.0058 (15)	−0.0052 (13)	−0.0137 (14)
C4	0.0202 (13)	0.0257 (14)	0.0158 (12)	0.0085 (10)	−0.0025 (10)	−0.0045 (10)
C5	0.0190 (13)	0.0303 (15)	0.0173 (13)	0.0093 (11)	0.0031 (10)	0.0012 (11)
C6	0.0363 (18)	0.043 (2)	0.0228 (15)	0.0080 (15)	0.0072 (13)	0.0047 (14)
C7	0.0181 (13)	0.0259 (16)	0.0207 (16)	−0.0041 (11)	0.0036 (12)	0.0049 (13)
C8	0.0139 (13)	0.0169 (15)	0.0157 (14)	−0.0014 (10)	−0.0005 (11)	−0.0009 (12)
C9	0.0117 (11)	0.0151 (12)	0.0142 (11)	0.0001 (9)	−0.0005 (9)	−0.0038 (9)
C10	0.0106 (11)	0.0166 (12)	0.0161 (12)	−0.0021 (9)	0.0005 (9)	−0.0042 (9)
C11	0.0065 (11)	0.0168 (13)	0.0135 (14)	−0.0025 (9)	0.0012 (10)	−0.0017 (11)
C12	0.0136 (13)	0.0244 (15)	0.0152 (14)	−0.0036 (11)	−0.0040 (11)	−0.0055 (12)
C13	0.0214 (13)	0.0216 (14)	0.0140 (12)	−0.0088 (10)	−0.0044 (10)	−0.0022 (10)
C14	0.0285 (15)	0.0325 (16)	0.0190 (13)	−0.0058 (12)	0.0027 (11)	−0.0060 (12)
C15	0.0361 (18)	0.063 (3)	0.0220 (15)	−0.0132 (17)	0.0078 (13)	−0.0121 (16)
C16	0.050 (2)	0.067 (3)	0.0170 (14)	−0.034 (2)	0.0022 (14)	−0.0008 (16)
C17	0.082 (3)	0.037 (2)	0.025 (2)	−0.030 (2)	−0.016 (2)	0.0138 (18)
C18	0.047 (2)	0.027 (2)	0.0249 (19)	−0.0061 (16)	−0.0111 (16)	0.0009 (15)
N1	0.0104 (10)	0.0143 (10)	0.0140 (10)	−0.0030 (8)	0.0001 (8)	−0.0017 (8)
O1	0.0196 (9)	0.0250 (10)	0.0139 (8)	−0.0045 (7)	−0.0015 (7)	−0.0060 (7)
O2	0.0065 (8)	0.0321 (11)	0.0197 (9)	0.0000 (7)	−0.0006 (7)	−0.0073 (8)
F1	0.101 (2)	0.082 (2)	0.0154 (10)	0.0127 (17)	0.0165 (12)	−0.0005 (11)
Br1	0.01948 (11)	0.01430 (11)	0.03333 (14)	−0.00276 (7)	−0.00045 (8)	−0.00482 (8)

### Geometric parameters (Å, º)

**Table:**

C1—C2	1.375 (7)	C10—O2	1.421 (3)
C1—C6	1.360 (6)	C11—H11A	0.9900
C1—F1	1.372 (4)	C11—H11B	0.9900
C2—H2	0.9500	C11—N1	1.502 (3)
C2—C3	1.392 (6)	C12—H12A	0.9900
C3—H3	0.9500	C12—H12B	0.9900
C3—C4	1.396 (4)	C12—C13	1.503 (4)
C4—C5	1.387 (5)	C12—N1	1.513 (4)
C4—O1	1.377 (3)	C13—C14	1.392 (4)
C5—C6	1.403 (4)	C13—C18	1.393 (5)
C5—C7	1.508 (5)	C14—H14	0.9500
C6—H6	0.9500	C14—C15	1.390 (4)
C7—H7A	0.9900	C15—H15	0.9500
C7—H7B	0.9900	C15—C16	1.367 (6)
C7—C8	1.525 (4)	C16—H16	0.9500
C8—H8A	0.9900	C16—C17	1.384 (7)
C8—H8B	0.9900	C17—H17	0.9500
C8—C9	1.511 (4)	C17—C18	1.399 (7)
C9—H9	1.0000	C18—H18	0.9500
C9—C10	1.527 (3)	N1—H1A	0.9900
C9—O1	1.446 (3)	N1—H1B	0.9900
C10—H10	1.0000	O2—H2A	0.8400
C10—C11	1.524 (4)		
			
C6—C1—C2	123.4 (3)	O2—C10—H10	107.5
C6—C1—F1	118.2 (4)	O2—C10—C11	112.2 (2)
F1—C1—C2	118.4 (4)	C10—C11—H11A	109.6
C1—C2—H2	121.0	C10—C11—H11B	109.6
C1—C2—C3	118.1 (4)	H11A—C11—H11B	108.1
C3—C2—H2	121.0	N1—C11—C10	110.3 (2)
C2—C3—H3	120.3	N1—C11—H11A	109.6
C2—C3—C4	119.4 (4)	N1—C11—H11B	109.6
C4—C3—H3	120.3	H12A—C12—H12B	108.1
C5—C4—C3	121.5 (3)	C13—C12—H12A	109.6
O1—C4—C3	115.2 (3)	C13—C12—H12B	109.6
O1—C4—C5	123.2 (3)	C13—C12—N1	110.4 (2)
C4—C5—C6	118.2 (3)	N1—C12—H12A	109.6
C4—C5—C7	121.0 (2)	N1—C12—H12B	109.6
C6—C5—C7	120.8 (3)	C14—C13—C12	120.2 (3)
C1—C6—C5	119.4 (4)	C14—C13—C18	119.1 (3)
C1—C6—H6	120.3	C18—C13—C12	120.7 (3)
C5—C6—H6	120.3	C13—C14—H14	119.6
C5—C7—H7A	109.7	C15—C14—C13	120.7 (3)
C5—C7—H7B	109.7	C15—C14—H14	119.6
C5—C7—C8	109.8 (3)	C14—C15—H15	119.9
H7A—C7—H7B	108.2	C16—C15—C14	120.3 (4)
C8—C7—H7A	109.7	C16—C15—H15	119.9
C8—C7—H7B	109.7	C15—C16—H16	120.2
C7—C8—H8A	109.9	C15—C16—C17	119.7 (3)
C7—C8—H8B	109.9	C17—C16—H16	120.2
H8A—C8—H8B	108.3	C16—C17—H17	119.5
C9—C8—C7	109.1 (3)	C16—C17—C18	120.9 (4)
C9—C8—H8A	109.9	C18—C17—H17	119.5
C9—C8—H8B	109.9	C13—C18—C17	119.3 (4)
C8—C9—H9	108.7	C13—C18—H18	120.4
C8—C9—C10	115.5 (2)	C17—C18—H18	120.4
C10—C9—H9	108.7	C11—N1—C12	112.5 (2)
O1—C9—C8	110.5 (2)	C11—N1—H1A	109.1
O1—C9—H9	108.7	C11—N1—H1B	109.1
O1—C9—C10	104.45 (19)	C12—N1—H1A	109.1
C9—C10—H10	107.5	C12—N1—H1B	109.1
C11—C10—C9	110.3 (2)	H1A—N1—H1B	107.8
C11—C10—H10	107.5	C4—O1—C9	115.5 (2)
O2—C10—C9	111.7 (2)	C10—O2—H2A	109.5
			
C1—C2—C3—C4	−0.1 (5)	C10—C9—O1—C4	−171.2 (2)
C2—C1—C6—C5	−0.1 (6)	C10—C11—N1—C12	−173.5 (2)
C2—C3—C4—C5	−0.6 (5)	C12—C13—C14—C15	−179.8 (3)
C2—C3—C4—O1	179.8 (3)	C12—C13—C18—C17	−179.5 (4)
C3—C4—C5—C6	0.9 (4)	C13—C12—N1—C11	178.6 (2)
C3—C4—C5—C7	−178.8 (3)	C13—C14—C15—C16	−1.5 (5)
C3—C4—O1—C9	−166.3 (2)	C14—C13—C18—C17	−0.4 (5)
C4—C5—C6—C1	−0.6 (5)	C14—C15—C16—C17	1.3 (6)
C4—C5—C7—C8	17.2 (4)	C15—C16—C17—C18	−0.6 (6)
C5—C4—O1—C9	14.1 (4)	C16—C17—C18—C13	0.1 (7)
C5—C7—C8—C9	−47.7 (3)	C18—C13—C14—C15	1.0 (5)
C6—C1—C2—C3	0.4 (6)	N1—C12—C13—C14	72.8 (3)
C6—C5—C7—C8	−162.5 (3)	N1—C12—C13—C18	−108.0 (3)
C7—C5—C6—C1	179.1 (3)	O1—C4—C5—C6	−179.5 (3)
C7—C8—C9—C10	−177.6 (2)	O1—C4—C5—C7	0.8 (4)
C7—C8—C9—O1	64.1 (3)	O1—C9—C10—C11	−168.4 (2)
C8—C9—C10—C11	70.0 (3)	O1—C9—C10—O2	66.1 (2)
C8—C9—C10—O2	−55.4 (3)	O2—C10—C11—N1	−52.0 (3)
C8—C9—O1—C4	−46.4 (3)	F1—C1—C2—C3	−179.5 (3)
C9—C10—C11—N1	−177.2 (2)	F1—C1—C6—C5	179.8 (3)

### Hydrogen-bond geometry (Å, º)

**Table:**

*D*—H···*A*	*D*—H	H···*A*	*D*···*A*	*D*—H···*A*
N1—H1*A*···Br1^i^	0.99	2.40	3.306 (2)	152
N1—H1*B*···Br1^ii^	0.99	2.30	3.258 (2)	162
O2—H2*A*···Br1^i^	0.84	2.47	3.2198 (19)	149

Symmetry codes: (i) *x*, *y*−1, *z*−1; (ii) *x*, *y*, *z*−1.
